# Mechanical power density, spontaneous breathing indexes, and prolonged weaning failure: a prospective cohort study

**DOI:** 10.1038/s41598-024-67237-w

**Published:** 2024-07-15

**Authors:** Alessandro Ghiani, Swenja Walcher, Azal Lutfi, Joanna Paderewska, Simon Ulrich Jaeger, Nikolaus Kneidinger, Stephanie Susanne Stecher, Franziska Christina Trudzinski, Claus Neurohr

**Affiliations:** 1 Department of Pulmonology and Respiratory Medicine, Lung Center Stuttgart – Schillerhoehe Lung Clinic, affiliated to the Robert-Bosch-Hospital GmbH, Auerbachstrasse 110, 70376 Stuttgart, Germany; 2 Department of Medicine V, LMU University Hospital, LMU Munich, Comprehensive Pneumology Center, Member of the German Center for Lung Research (DZL), Munich, Germany; 3 Division of Pulmonology, Department of Internal Medicine, Medical University of Graz, Graz, Austria; 4 Department of Medicine II, LMU University Hospital, LMU Munich, Munich, Germany; 5 Department of Pneumology and Critical Care Medicine, Thoraxklinik, Translational Lung Research Center Heidelberg (TLRC-H), German Center for Lung Research (DZL), University of Heidelberg, Heidelberg, Germany; 6 Comprehensive Pneumology Center, Member of the German Center for Lung Research (DZL), Munich, Germany

**Keywords:** Mechanical ventilation, Prolonged weaning, Spontaneous breathing trial, Tracheostomy, Mechanical power, Biomarkers, Risk factors

## Abstract

A prospective observational study comparing mechanical power density (MP normalized to dynamic compliance) with traditional spontaneous breathing indexes (e.g., predicted body weight normalized tidal volume [VT/PBW], rapid shallow breathing index [RSBI], or the integrative weaning index [IWI]) for predicting prolonged weaning failure in 140 tracheotomized patients. We assessed the diagnostic accuracy of these indexes at the start and end of the weaning procedure using ROC curve analysis, expressed as the area under the receiver operating characteristic curve (AUROC). Weaning failure occurred in 41 out of 140 patients (29%), demonstrating significantly higher MP density (6156 cmH_2_O^2^/min [4402–7910] vs. 3004 cmH_2_O^2^/min [2153–3917], *P* < 0.01), lower spontaneous VT/PBW (5.8 mL*kg^−1^ [4.8–6.8] vs. 6.6 mL*kg^−1^ [5.7–7.9], *P* < 0.01) higher RSBI (68 min^−1^*L^−1^ [44–91] vs. 55 min^−1^*L^−1^ [41–76], *P* < 0.01) and lower IWI (41 L^2^/cmH_2_O*%*min*10^−3^ [25–72] vs. 71 L^2^/cmH_2_O*%*min*10^-3^ [50–106], *P* < 0.01) and at the end of weaning. MP density was more accurate at predicting weaning failures (AUROC 0.91 [95%CI 0.84–0.95]) than VT/PBW (0.67 [0.58–0.74]), RSBI (0.62 [0.53–0.70]), or IWI (0.73 [0.65–0.80]), and may help clinicians in identifying patients at high risk for long-term ventilator dependency.

## Introduction

Determining a patient’s spontaneous breathing ability is one of the most challenging tasks during prolonged weaning. Patients unable to maintain autonomic breathing usually remain ventilator-dependent over the long term, resulting in reduced health-related quality of life^1^ and high mortality rates^2^. Previous studies on weaning predictors have focused on variables associated with spontaneous breathing and indexes derived from esophageal pressure measurements (e.g., diaphragmatic tension-time index)^3,4^, quantifying respiratory muscle workload, a key factor in prolonged weaning failure. However, most of these single parameters (e.g., respiratory rate, tidal volume) are not accurate at predicting weaning outcomes, most likely because they do not reflect weaning failure’s underlying pathophysiology, and the complexity of the procedure prevents esophageal catheters from being integrated into daily routines.

Mechanical power (MP) is the displacement work provided by the ventilator each minute^5^, with ventilatory variables determining MP adjusted by clinicians to maintain patient oxygenation and decarboxylation (e.g., positive end-expiratory pressure, minute ventilation). As such, MP could serve to estimate respiratory muscle workload during spontaneous, unassisted breathing in an indirect manner. There is preliminary evidence that MP normalized to dynamic lung-thorax compliance, essentially a pressure-rate index measuring the intensity of mechanical stress exerted on the respiratory system and consistent with MP density, may help separate patients experiencing weaning failure or success^6,7^. However, MP density has not yet been compared to spontaneous breathing indexes for predicting a patient’s autonomic breathing ability following prolonged mechanical ventilation.

The study objectives were (1) to evaluate MP density’s predictive performance compared to traditional breathing indexes assessed during a protocolized spontaneous breathing trial (SBT) regarding weaning failure in prolonged ventilated, tracheotomized patients, (2) to analyze the interactions between ventilatory and spontaneous breathing indexes reflecting respiratory muscle workload, and (3) to correlate these indexes with P_a_CO_2_ during spontaneous breathing at the end of weaning.

## Methods

We conducted a prospective, single-center observational cohort study at a German national weaning center. The local institutional review board for human studies approved the project (Ethics Committee of the State Chamber of Physicians of Baden-Wuerttemberg, Germany, file number F-2021–118), which was performed following the Declaration of Helsinki. All patients or their legal representatives provided written informed consent.

### Patient selection

**Figure 1:**
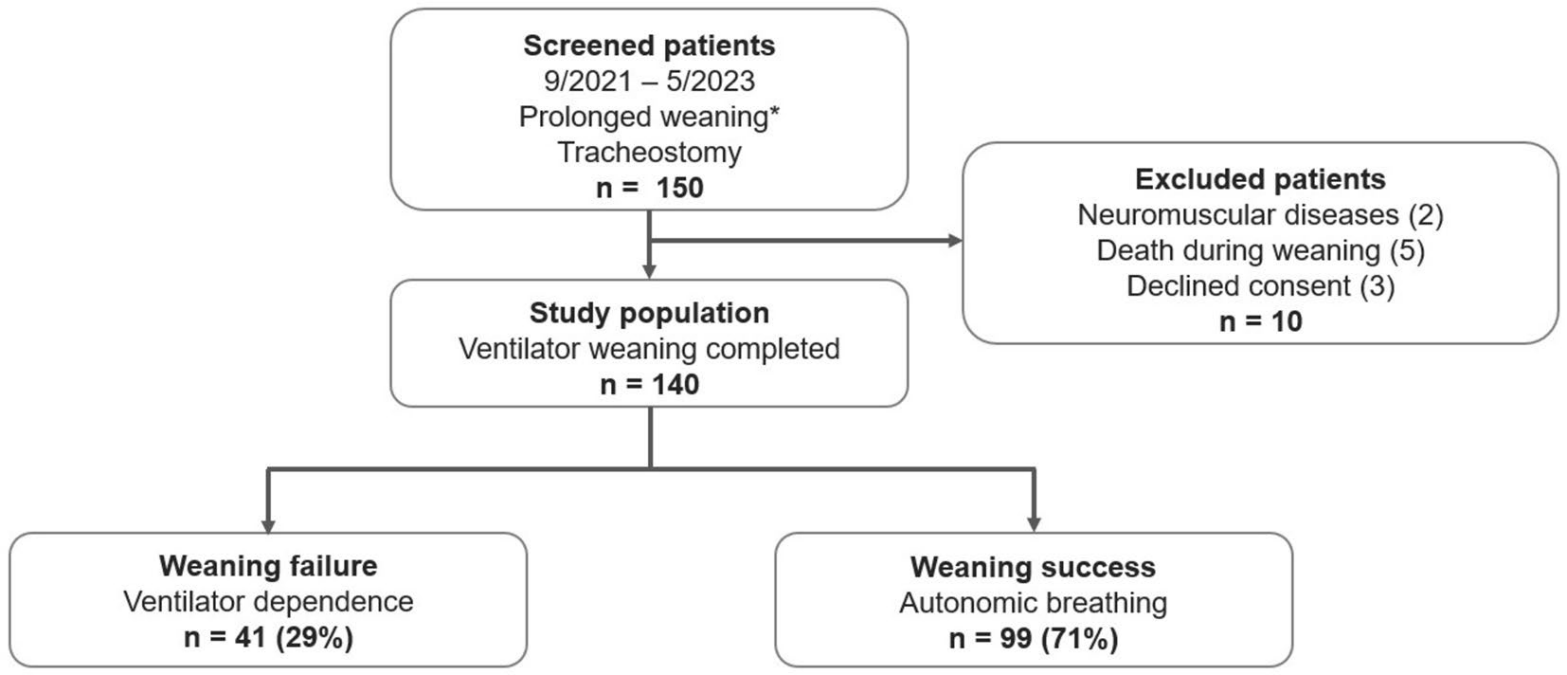
Patient flow diagram.*Refers to the WIND criteria for prolonged weaning^8^.

We included tracheotomized subjects referred from ICUs across Germany to our center between September 2021 and May 2023 for “*prolonged weaning*” according to the WIND criteria [Fig. 1]. By definition, these patients were still on mechanical ventilation 7 days after their first separation attempt, which in most cases was an SBT with or without subsequently attempted extubation in the referring ICU^8^. The referring facilities included both external and internal ICUs in a 50:50 ratio. Exclusion criteria were a diagnosis of neuromuscular disease, death during weaning, and declined consent for participation^6^.

### Data collection

**Figure 2:**
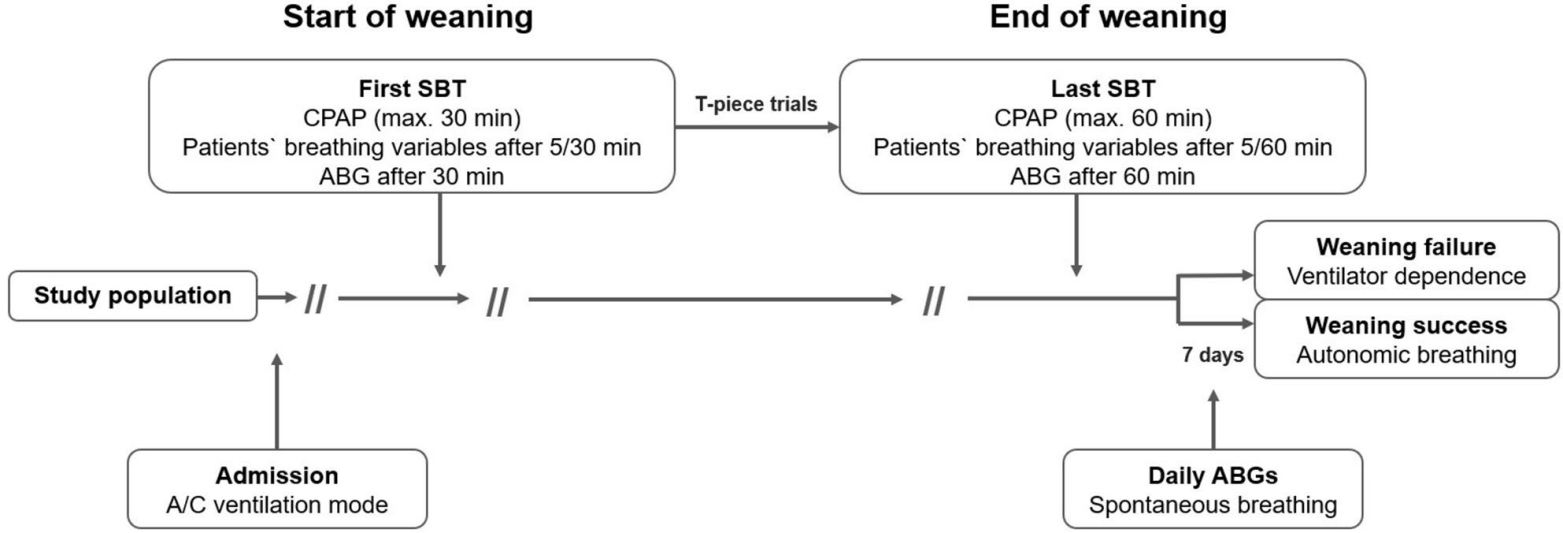
Study flow and timeline for assessing ventilatory and breathing indexes used to predict weaning outcome. *SBT* spontaneous breathing trial, *CPAP* continuous positive airway pressure, *ABG* arterial blood gas analysis.

Baseline characteristics, including demographics, clinical features, and comorbidities, were collected on patients upon weaning center admission. We created a protocol for the documentation of the first (start of weaning) and last (end of weaning) SBT [Supplementary file 1] and summarized the results of prolonged weaning. Finally, a clinical follow-up was conducted up to 7 days after weaning completion to determine if progressive hypercapnia developed during spontaneous breathing, requiring resumption of mechanical ventilation and indicating weaning failure [Fig. 2].

### Ventilator weaning process

Upon weaning center admission, pressure-controlled, assist-control (A/C mode, without setting a target volume) mechanical ventilation (Elisa 600, Löwenstein Medical, Bad Ems, Germany) was used on all patients to unload the respiratory pump effectively during both an assisted and controlled breath. With this mode, an assisted breath does not necessarily indicate a high level of spontaneous activity. When the ventilator is triggered with minimal effort, it delivers a breath similar to controlled ventilation (passive insufflation occurs)^9^.

As described previously, a standardized method of ventilator liberation was employed as soon as clinical screening criteria for weaning readiness were met, including inspired oxygen fraction (F_i_O_2_) ≤ 0.4, positive end-expiratory pressure (PEEP) ≤ 8 cmH_2_O, stable hemodynamics without vasopressors or inotropic agents, and normocapnia with mechanical ventilation^6^. According to protocol, ventilator weaning always starts with a 30 min SBT. These weaning trials (using a T-piece) are usually conducted once daily, and their duration is typically extended by 2–3 h per day, aiming to achieve sustained autonomic breathing. Following ventilator weaning, patients were evaluated for decannulation, with those demonstrating persistent ventilatory failure (hypercapnia) transitioning to non-invasive ventilation whenever possible^10^.

### Spontaneous breathing trials

Analyses were conducted on a protocolized SBT at the start (first SBT) and end of weaning (last SBT), which used continuous positive airway pressure (CPAP) to assess patients` spontaneous breathing variables.

As a first step, patients were placed in the semi-recumbent position while mechanically ventilated, “*ventilatory variables*” were recorded, and an arterial blood gas analysis (ABG) was performed. Next, we applied CPAP, which was set at the same level as PEEP during A/C ventilation. CPAP breathing was limited to 30/60 min at first/last SBTs [Fig. 2], with patients’ “*spontaneous breathing variables*” recorded from the ventilators display at the weaning trials beginning (after 5 min) and completion (after 30/60 min of the first/last SBT). Another ABG was performed at the end of each trial.

### Ventilatory variables and indexes

According to protocol, ventilatory variables and ABG measurements were recorded immediately before the SBT. Variables collected included F_i_O_2_, respiratory rate (RR), tidal volume (VT), peak inspiratory airway pressure (P_max_), and PEEP, with the following parameters calculated: ∆P_aw_ (dynamic driving pressure, defined as P_max—_PEEP), dynamic lung-thorax compliance (LTC_dyn_, defined as VT/∆P_aw_), ventilatory ratio (VR; a measure of ventilation efficiency, correlating with the pulmonary dead-space fraction)^11^, and MP utilizing a simplified formula for pressure-control ventilation^12^.

### Mechanical power and MP density

With each mechanical breath, the pressure required to inflate the lungs and expand the thoracic cage is used to overcome the resistance of the airways (dissipating heat) and the elastance of the lungs and chest wall. As such, mechanical energy is the displacement work (pressure times volume) the ventilator provides per breath as determined by applied P_max_ (including PEEP) and VT during pressure-controlled ventilation^12^. Concerning elastance, given that the lungs and thorax expand predominantly elastically, most of the energy is stored with each breath, which ultimately drives expiration (through respiratory systems’ elastic recoil pressure)^13^.

The same mechanical energy per breath can be achieved with various combinations of P_max_ and VT, as determined by the amount of aerated lung tissue, chest wall elastance, and airway resistance. Dynamic respiratory system compliance provides a simple proxy for all these factors. Normalizing energy per breath to dynamic compliance estimates the relative contributions of P_max_ and VT to a particular energy level. When compliance is low, higher pressure is required to achieve the same amount of energy as when compliance is high, in which, despite lower pressures, greater tidal volumes are generated. In other words, at a given tidal volume (strain), lower compliance results in more energy being transferred per breath, resulting in higher (strain) “*energy density*”. Given this, energy density mainly represents the stored energy per unit of (tidal) volume resulting from the body’s elastic deformation (resistive-elastic unicompartmental model)^14^.

Multiplying the energy per breath by the respiratory rate results in mechanical power, the amount of energy transferred to the respiratory system per minute. The “*MP density*” is calculated by multiplying the energy density—energy per breath normalized to dynamic compliance—with the respiratory rate, quantifying the energy transferred per minute with respect to the volume applied per minute.

$$LTC_{dyn} - MP \, \left[ {cmH_{2} O^{2} /min} \right] \, = \, RR \, * \, P_{max} * \, \Delta P_{aw} .$$

This index measures the intensity of mechanical stress placed on the respiratory system, determined by peak airway pressure, dynamic driving pressure, and the frequency of their application (pressure application per time), quantifying the time rate (or speed) of energy transfer per unit of volume displaced. It has also been referred to as the “*specific MP*”, which is the power in relation to the ventilated lung volume^15^.

In a subsequent step, LTC_dyn_-MP was normalized to P_a_CO_2_ similarly to the corrected minute ventilation^16^ to approximate the MP density required for adequate decarboxylation. We used 45 mmHg as the target P_a_CO_2_ originally termed the respiratory system Power index^6^. For convenience, LTC_dyn_-MP and its P_a_CO_2_ normalized values will be referred to as “*MP density indexes*” throughout the manuscript. Detailed descriptions and equations for all computed variables are provided in the online supplement [Supplementary file 1].

### Spontaneous breathing variables and indexes

We collected the following variables during CPAP breathing, as per protocol, at the weaning trials onset (after 5 min) and completion (after 30/60 min of first/last SBT): F_i_O_2_, peripheral oxygen saturation measured with pulse oximetry (S_p_O_2_), respiratory rate, tidal volume, minute ventilation, and the ratio of inspiratory to expiratory time. We calculated the following parameters using these variables: tidal volume normalized to the predicted body weight (VT/PBW), rapid shallow breathing index (RSBI; also referred to as frequency-to-tidal volume ratio)^17^, and a modified integrative weaning index (IWI modified; using LTC_dyn_ instead of the static respiratory system compliance)^18^.

### Classification of weaning outcomes

At the end of the ventilator weaning process, patients were categorized into two groups exclusively based on spontaneous breathing abilities: weaning failure and success^6^. Weaning failure is defined as “*long-term ventilator dependence*” due to persistent ventilatory failure with transitioning to domiciliary ventilation by face mask or tracheostomy tube. Ventilatory failure describes recurrent hypercapnia (P_a_CO_2_ > 45 mmHg) during daily weaning trials (observed on at least two consecutive days), preventing the extension of spontaneous unassisted breathing or persistent hypercapnia (on at least two successive occasions) occurring within seven days after weaning completion. These patients usually remain ventilator-attached at discharge. Conversely, the definition of successful weaning is sustained “*autonomic breathing*” (≥ 7 days) without concomitant signs of ventilatory failure (hypercapnia) after weaning completion (median P_a_CO_2_ ≤ 45.0 mmHg derived from the highest measured P_a_CO_2_ on each of the seven days), determined by the last day on which the patient was ventilated. These patients typically remain ventilator-detached at hospital discharge.

### Primary and secondary study outcomes

The study`s primary objective was to compare MP density (before SBT) with spontaneous breathing indexes (during SBT) regarding prolonged weaning failure prediction. Secondary outcomes included an analysis of the interaction between ventilatory and spontaneous breathing indexes reflecting respiratory muscle workload and the parameters` correlation to spontaneous breathing P_a_CO_2_.

### Statistical analysis and sample size determination

Descriptive and frequency statistics summarized patients` baseline demographics, clinical characteristics, and comorbidities. A Chi-square or Fisher’s exact test was used when comparing categorical variables. Depending on the continuous variables` homogeneity of variance, determined by the Kolmogorov-Smirnov normality test, differences were analyzed through Student’s *t*-test or Mann-Whitney *U*-test.

First, we performed bivariate comparisons of ventilatory and spontaneous breathing indexes between weaning failure and success patients at the onset and end of the weaning process. Next, we assessed the ability of these indexes to predict prolonged weaning failure by performing a receiver operating characteristic (ROC) curve analysis in the entire study population, with diagnostic accuracy expressed as the area under the ROC curve (AUROC). Moreover, we performed 2-time repeated, fivefold cross-validation to evaluate the internal validity of these indexes. The resulting diagnostic performance of each parameter was expressed as sensitivity, specificity, positive/negative predictive value, accuracy, positive/negative likelihood ratio, diagnostic odds ratio (DOR), F_1_ score, and Matthews` correlation coefficient (MCC). Rank correlations and linear regression analyses were conducted to determine interactions between ventilatory (e.g., dynamic compliance, power density) and spontaneous breathing indexes (e.g., RSBI) reflecting respiratory muscle workload. Finally, we correlated indexes assessed at last SBT with median spontaneous breathing P_a_CO_2_ at the end of weaning (during follow-up) using Spearmans` correlation coefficient (*ρ*).

We conducted sensitivity analyses by redefining the P_a_CO_2_ threshold for ventilatory failure (to > 50 mmHg) or restricting the analysis to patients without COVID-19 pneumonia as the leading cause of prolonged ventilation, as their respiratory mechanics may differ from those of other respiratory failure patients^19^. Furthermore, we reanalyzed rank correlations (*ρ*) between parameters and P_a_CO_2_ limited to weaning success patients.

Based on an α-level (type-I error) of 0.05 with power (1‒β) of 80%, an expected prevalence of prolonged weaning failure of 40%, and an expected MP density`s AUROC of 0.80–0.90 for prolonged weaning failure prediction (at the end of weaning)^6^, we calculated a sample size of about 140 patients for detecting AUROC differences of at least 10% between power density and spontaneous breathing indexes.

We performed two-tailed tests; statistical significance was indicated by *P* < 0.05. The analyses were conducted with MedCalc® statistical software v20.305 (MedCalc Software Ltd, Ostend, Belgium; http://www.medcalc.org; 2023).

### Ethics approval and consent to participate

The study was approved by the local institutional review board for human studies (Ethics Committee of the State Chamber of Physicians of Baden-Wuerttemberg, Germany, approval number F–2021–118) and performed following the Declaration of Helsinki. Written informed consent was obtained from all patients or a legal representative.

## Results

Of 150 consecutive patients screened between September 2021 and May 2023, 140 (93%) were included in the analyses. The reasons for exclusion were a confirmed diagnosis of neuromuscular disease in two patients, five patients died during weaning, and three patients declined consent [Fig. 1]. Clinical characteristics differed between patients with weaning failure and success, mainly regarding gender and associated PBW, smoking history, pre-existing domiciliary non-invasive ventilation, and chronic obstructive pulmonary disease (COPD) as comorbidity [Table 1].

**Table 1:** Clinical characteristics on admission to the weaning center—comparison of patients with weaning failure and success.

Clinical characteristics	All patients (n = 140)	Weaning failure (n = 41)	Weaning success (n = 99)	*P* value^*a*^
Age (years)	68 (59–74)	69 (59–74)	68 (58–74)	0.491^*c*^
Female gender	48 (34)	19 (46)	29 (29)	0.054^*d*^
Predicted body weight (kg)	66 (57–75)	64 (54–71)	70 (62–75)	**0.032**^***c***^
Body mass index (kg/m^2^)	27 (24–31)	28 (22–31)	27 (25–31)	0.719^*c*^
* Obesity (BMI* ≥ *30 kg/m*^*2*^*)*	43 (31)	13 (32)	30 (30)	0.870^*d*^
Smoking history	45 (32)	23 (56)	22 (22)	** < 0.01**^***d***^
APACHE-II (points)	15 (11–17)	15 (12–17)	15 (11–17)	0.708^*b*^
Albumin (g/dL)	2.6 (2.3–3.0)	2.7 (2.5–3.2)	2.6 (2.2–2.9)	0.050^*b*^
Pre-existing domiciliary NIV	5 (4)	4 (10)	1 (1)	**0.026**^***e***^
Percutaneous tracheostomy	107 (76)	30 (73)	77 (78)	0.560
Ventilator days on admission	23 (16–34)	21 (14–34)	23 (16–34)	0.437^*c*^
Intubation to tracheostomy (days)	13 (9–18)	13 (8–17)	13 (9–19)	0.400^*c*^
Extracorporeal lung support	20 (14)	4 (10)	16 (16)	0.326^*d*^
Reason for mechanical ventilation				
Pneumonia	52 (37)	13 (32)	39 (39)	0.393^*d*^
* SARS-CoV-2 infection*	30 (21)	6 (15)	24 (24)	0.209^*d*^
Surgery	49 (35)	12 (29)	37 (37)	0.362^*d*^
Cardiopulmonary resuscitation	19 (14)	4 (10)	15 (15)	0.398^*d*^
Acute exacerbation of COPD	9 (6)	9 (22)	0 (0.0)	** < 0.01**^***e***^
Sepsis (extrapulmonary)	7 (5)	1 (2)	6 (6)	0.673^*e*^
Other	4 (3)	2 (5)	2 (2)	0.580^*e*^
Comorbidities				
Charlson comorbidity index (points)	5 (3–6)	5 (4–6)	4 (3–6)	0.215^*c*^
Renal insufficiency	46 (33)	13 (32)	33 (33)	0.853^*d*^
Coronary artery disease	41 (29)	13 (32)	28 (28)	0.686^*d*^
Diabetes mellitus	35 (25)	11 (27)	24 (24)	0.749^*d*^
COPD	25 (18)	18 (44)	7 (7)	** < 0.01**^***d***^
Chronic heart failure	16 (11)	3 (7)	13 (13)	0.327^*d*^
Hepatopathy	9 (6)	1 (2)	8 (8)	0.283^*e*^
Malignancy	5 (4)	2 (5)	3 (3)	0.630^*e*^

Continuous variables are presented as median (– interquartile range [IQR]); categorical variables are presented as numbers (%).

*BMI* body mass index, *APACHE-II* Acute Physiology and Chronic Health Evaluation II score, *NIV* non-invasive ventilation, *SARS-CoV-2* severe acute respiratory syndrome-Coronavirus 2, *COPD* chronic obstructive pulmonary disease.

^*a*^*P* value for differences between the weaning failure and success group.

^*b*^Student`s *t*-test.

^*c*^Mann-Whitney *U*-test.

^*d*^Chi-squared test.

^*e*^Fisher`s exact test.

Significant values are in bold.

Weaning failure occurred in 41 patients (29%) [Fig. 1, Supplementary file 1: Table S1], with significant differences between groups in ventilatory and spontaneous breathing variables at the first and last SBT [Supplementary file 1: Table S2-3].

### Ventilatory variables and indexes

**Figure 3:**
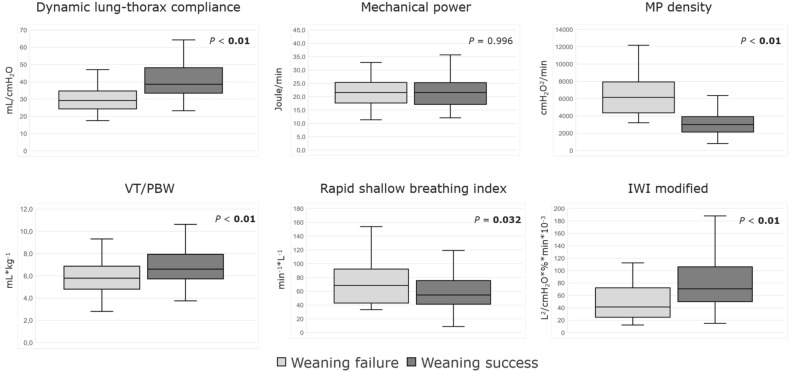
Between-group differences in selected ventilatory and breathing indexes at the end of weaning (last SBT). Comparison of selected ventilatory and spontaneous breathing indexes between weaning failure and success patients at the end of weaning (last SBT), as illustrated by Box-Whiskers plots. MP density refers to the Power index_rs_^2.0^. *VT/PBW* tidal volume normalized to the predicted body weight, *IWI modified* modified integrative weaning index.

During mechanical ventilation before the onset of weaning, weaning failure patients showed lower dynamic compliance (median 32 mL/cmH_2_O with IQR [29–39] vs. 39 mL/cmH_2_O [33–47], *P* < 0.01) and higher P_a_CO_2_ (39 mmHg [35–43] vs. 33 mmHg [28–38], *P* < 0.01). Even though minute ventilation was lower in the failure group, these patients demonstrated significantly higher ventilatory ratios (1.65 [1.39–2.11] vs. 1.40 [1.26–1.73], *P* < 0.01). There was no difference in MP (21.6 Joule/min [17.8–26.3] vs. 22.3 Joule/min [19.1–27.4], *P* = 0.405), but MP density (measured by the Power index_rs_^2.0^) was consistently higher in weaning failures (5116 cmH_2_O^2^/min [3601–7020] vs. 3004 cmH_2_O^2^/min [2034–4676], *P* < 0.01) [Supplementary file 1: Table S2]. Analyzing ventilatory variables before the last SBT yielded similar results [Fig. 3, Supplementary file 1: Table S3].

### Spontaneous breathing variables and indexes

As weaning trials concluded, an increase in respiratory rate was apparent, accompanied by a drop in tidal volume, which was more prominent in the weaning failure group, resulting in significantly lower minute ventilation (9.7 L*min^−1^ [7.8–11.4] vs. 11.7 L*min^−1^ [9.5–13.9], *P* < 0.01), lower VT/PBW (5.8 mL*kg^−1^ [5.1–6.7] vs. 6.6 mL*kg^−1^ [6.0–7.9], *P* < 0.01), higher RSBI (69 min^−1^*L^−1^ [54–97] vs. 58 min^−1^*L^−1^ [43–73], *P* < 0.01), and lower IWI (43 L^2^/cmH_2_O*%*min*10^−3^ [31–58] vs. 62 L^2^/cmH_2_O*%*min*10^−3^ [46–91], *P* < 0.01) at the start of weaning [Supplementary file 1: Table S2]. The failure group also exhibited significantly higher P_a_CO_2_ (46 mmHg [42–51] vs. 36 mmHg [32–43], *P* < 0.01) [Supplementary file 1: Table S2]. Similar results were obtained from analyzing variables at the end of weaning [Fig. 3, Supplementary file 1: Table S3].

### Diagnostic accuracy of ventilatory and breathing indexes

**Figure 4:**
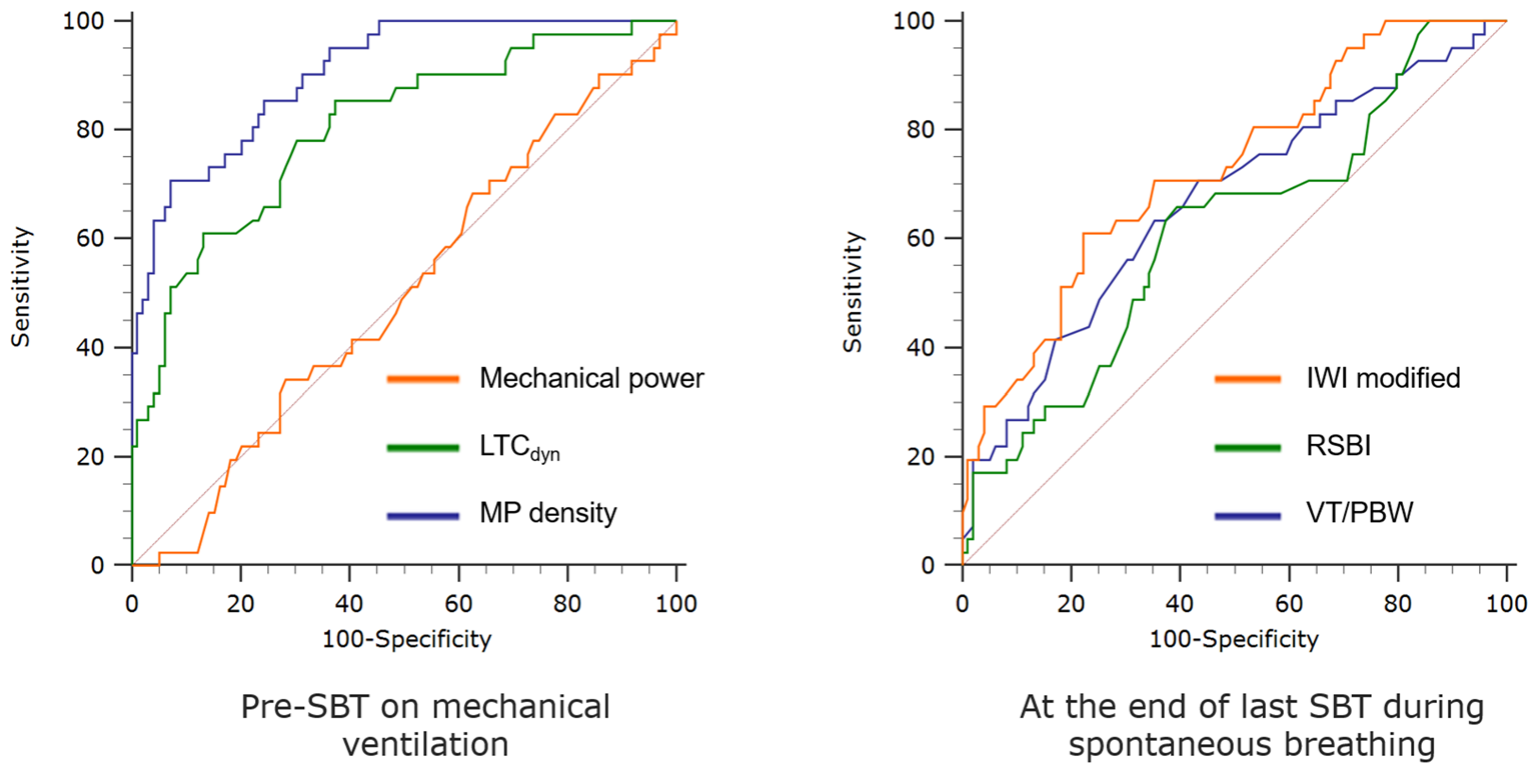
Comparison of ROC curves for selected ventilatory and breathing indexes used to predict weaning outcome at the end of weaning (last SBT). AUROC differed significantly between MP density (measured by the Power index_rs_^2.0^) and mechanical power (*P* < 0.01), LTC_dyn_ (*P* < 0.01), VT/PBW (*P* < 0.01), RSBI (*P* < 0.01), and IWI modified (*P* < 0.01). *ROC* receiver operating characteristic curve, *SBT* spontaneous breathing trial, *LTC*_*dyn*_ dynamic lung-thorax compliance, *IWI modified* modified integrative weaning index, *RSBI* rapid shallow breathing index, *VT/PBW* tidal volume normalized to the predicted body weight, *AUROC* area under the receiver operating characteristic curve.

MP density (as measured by the Power index_rs_^2.0^) demonstrated moderate diagnostic performance at the start of weaning (first SBT) (AUROC 0.76 [95%CI 0.68‒0.83]) and excellent accuracy at the end of weaning (last SBT) (AUROC 0.91 [0.84‒0.95], DOR 48, MCC 0.55) [Table 2, Supplementary file 1: Table S4]. According to AUROC measurements, MP density indexes at the start and end of weaning, as well as dynamic compliance, provided greater diagnostic accuracy in predicting weaning failure than spontaneous breathing indexes [Table 2, Fig. 4, Supplementary file 1: Table S4, Figure S1]. Similar results were obtained in the sensitivity analyses [Supplementary file 1: Table S5-6].

**Table 2:** Area under the ROC curve for each index analyzed at the start and end of weaning.

Ventilatory indexes (pre-SBT)	Start of weaning (first SBT)	*P* value	End of weaning (last SBT)	*P* value
Ventilatory ratio	0.66 (0.58–0.74)	** < 0.01**	0.68 (0.60–0.76)	** < 0.01**
LTC_dyn_ (mL/cmH_2_O)	0.71 (0.63–0.78)	** < 0.01**	0.80 (0.73–0.87)	** < 0.01**
Mechanical power (Joule/min)	0.55 (0.46–0.63)	0.401	0.50 (0.42–0.59)	0.996
MP density indexes				
* LTC*_*dyn*_*-MP (cmH*_*2*_*O*^*2*^*/min)*	0.67 (0.59–0.75)	** < 0.01**	0.79 (0.71–0.85)	** < 0.01**
* Power index*_*rs*_^*1.0*^* (cmH*_*2*_*O*^*2*^*/min)*	0.74 (0.66–0.81)	** < 0.01**	0.88 (0.82–0.93)	** < 0.01**
* Power index*_*rs*_^*2.0*^* (cmH*_*2*_*O*^*2*^*/min)*	0.76 (0.68–0.83)	** < 0.01**	0.91 (0.84–0.95)	** < 0.01**
Patients` breathing indexes (after 30/60 min)	Start of weaning (first SBT)	*P* value	End of weaning (last SBT)	*P* value
Tidal volume/PBW (mL/kg)	0.70 (0.62–0.78)	** < 0.01**	0.67 (0.58–0.74)	** < 0.01**
RSBI (min^−1^*L^−1^)	0.66 (0.58–0.74)	** < 0.01**	0.62 (0.53–0.70)	**0.030**
IWI modified (L^2^/cmH_2_O*%*min*10^−3^)	0.73 (0.65–0.80)	** < 0.01**	0.73 (0.65–0.80)	** < 0.01**

The accuracy of each ventilatory variable in predicting prolonged weaning failure is presented as the area under the ROC curve with 95% confidence intervals.

*SBT* spontaneous breathing trial, *P/F ratio* the ratio of partial pressure of oxygen to fraction of inspired oxygen, *LTC*_*dyn*_ dynamic lung-thorax compliance, *LTC*_*dyn*_*-MP* mechanical power normalized to dynamic lung-thorax compliance, *PBW* predicted body weight, *RSBI* rapid shallow breathing index, *IWI modified* modified integrative weaning index.

Significant values are in bold.

### Correlations between ventilatory and breathing indexes

**Figure 5:**
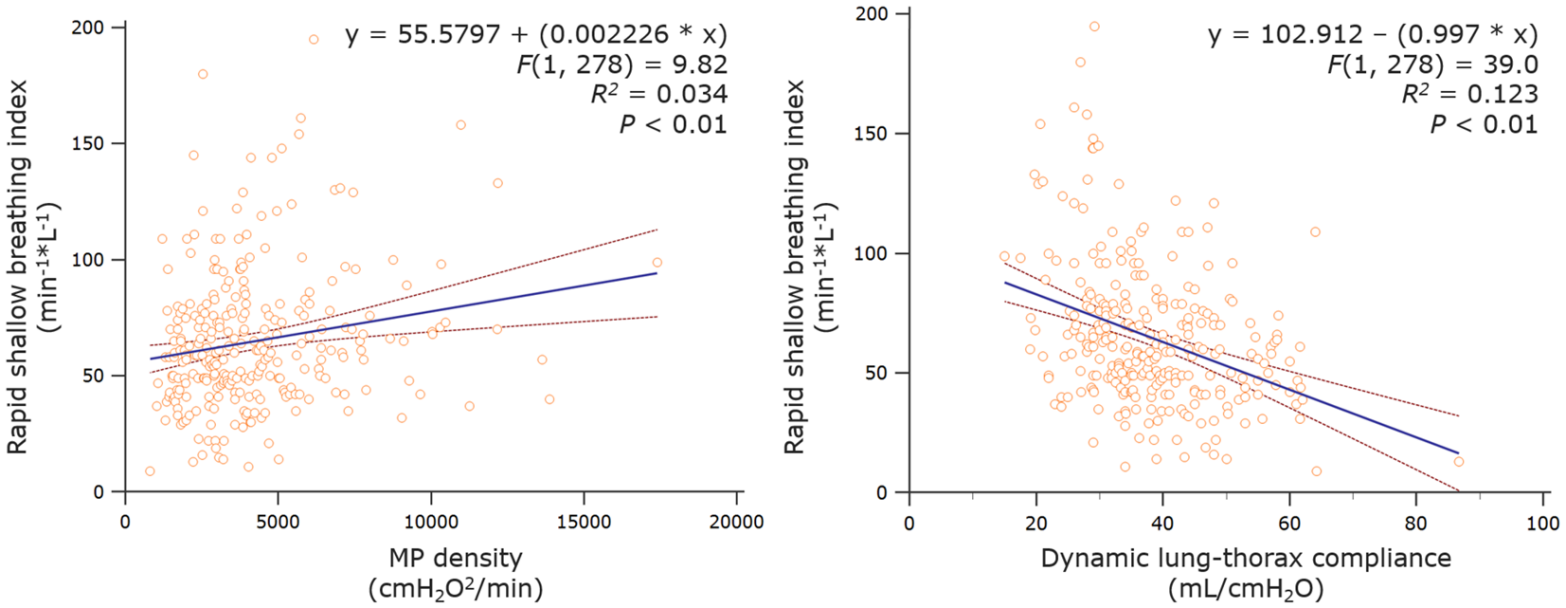
The rapid shallow breathing index as a function of MP density and dynamic lung-thorax compliance: linear regression analysis. An analysis of 280 RSBI and MP density (measured by the Power index_rs_^2.0^)/LTC_dyn_ measurements at the onset (first SBT) and end of weaning (last SBT). *RSBI* rapid shallow breathing index, *LTC*_*dyn*_ dynamic lung-thorax compliance.

Rank correlations and linear regression analyses were used to analyze interactions between ventilatory (e.g., MP density, dynamic compliance) and spontaneous breathing indexes (e.g., RSBI) reflecting respiratory muscle workload. Specifically, MP density (*ρ* = 0.18 [95%CI 0.06–0.29] for the Power index_rs_^2.0^; *P* < 0.01) and LTC_dyn_ (*ρ* =  − 0.33 [− 0.43 to − 0.22]; *P* < 0.01) revealed a significant correlation with the RSBI, as well as an independent relationship in linear regression analysis [Fig. 5].

### Correlations with spontaneous P_a_CO_2_

**Figure 6:**
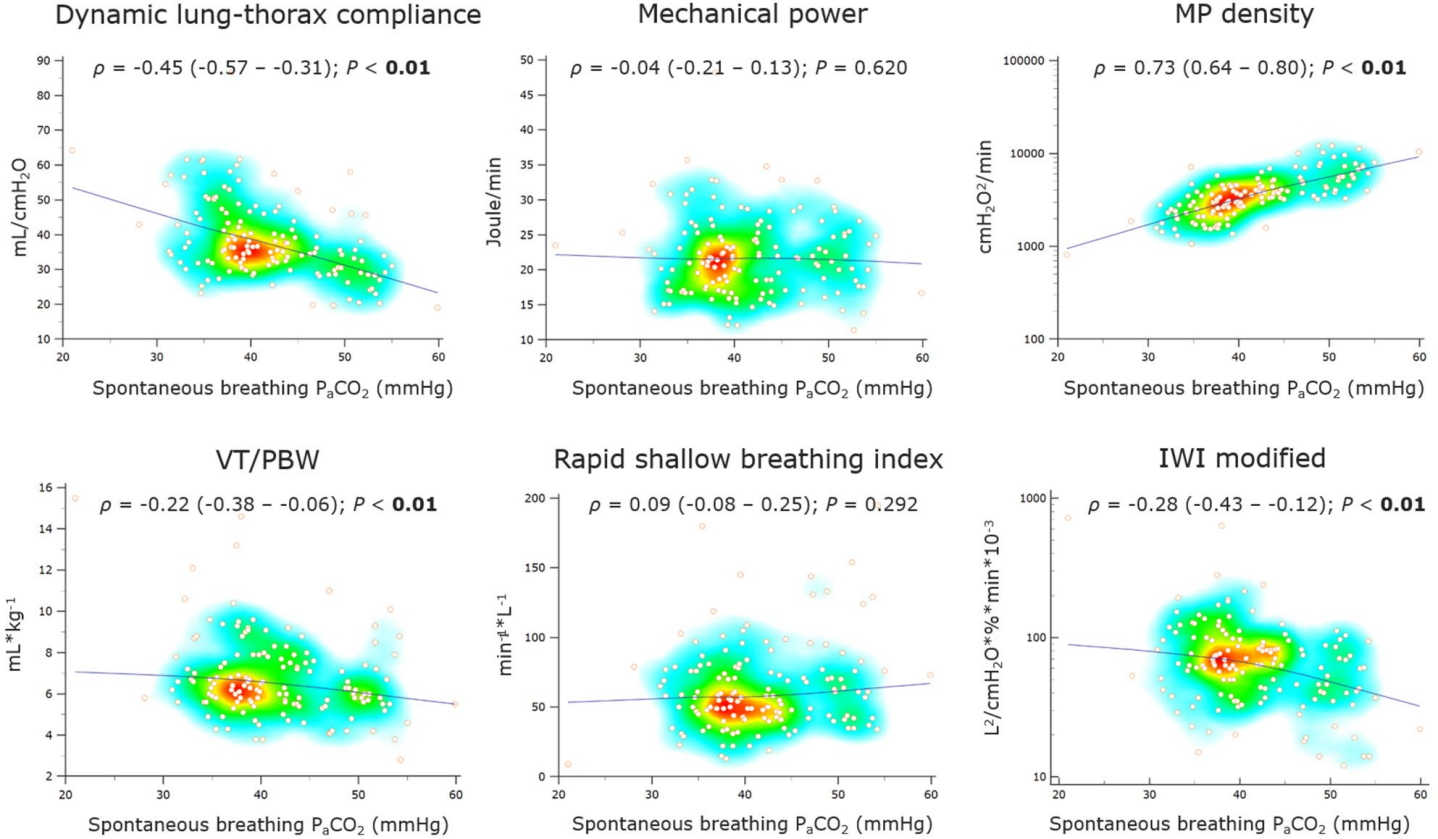
Correlations of selected ventilatory and breathing indexes at the end of weaning (last SBT) with spontaneous P_a_CO_2._ The heat map of Spearman`s correlation coefficients (*ρ*) with the LOESS (Local Regression Smoothing) trendline. Some Y axes have logarithmic scales. MP density refers to the Power index_rs_^2.0^. *ρ* Spearman`s correlation coefficient (with 95% confidence interval), *VT/PBW* tidal volume normalized to the predicted body weight, *IWI modified* modified integrative weaning index.

Most ventilatory and breathing indexes correlated moderately with spontaneous breathing P_a_CO_2_ at weaning completion [Fig. 6, Supplementary file 1: Table S7], whereas MP density had a strong correlation (*ρ* = 0.73 [95%CI 0.64–0.80] for the Power index_rs_^2.0^), maintained in a sensitivity analysis limited to 99 weaning success patients (*ρ* = 0.55 [95%CI 0.39–0.67]) [Supplementary file 1: Table S8].

### Exploratory analyses

COPD was significantly more prevalent among weaning failure patients. These patients mainly experienced significant differences in respiratory mechanics, MP density, and P_a_CO_2_ levels during mechanical ventilation and spontaneous breathing at the end of SBT [Supplementary file 1: Table S9].

## Discussion

Study results can be summarized as follows: Following prolonged weaning, weaning failures differed significantly in ventilatory and spontaneous breathing indexes compared to patients who sustained autonomic breathing (weaning success). While MP did not differ between groups, MP density indexes were significantly higher in patients experiencing weaning failure. These parameters were more accurate at predicting weaning failures than traditional indexes assessed during spontaneous breathing (e.g., VT/PBW, RSBI, or IWI), demonstrating significant correlations and independent associations with parameters reflecting respiratory workload (e.g., RSBI). A strong correlation was observed between MP density indexes and spontaneous breathing P_a_CO_2_ at the end of the weaning process.

With pressure-controlled ventilation, dynamic lung-thorax compliance determines tidal volume, minute ventilation, and MP at a given respiratory rate, P_max_, and PEEP^12^. If the latter three variables remain constant, a decrease in compliance will result in a linear decline in MP. Adjusting P_max_ to achieve the original (iso)-MP (at constant respiratory rate and PEEP) will result in reduced associated tidal volume and minute ventilation—compared to the values before compliance decreased—along with increased energy density and MP density. The reason is that every change in pressure is accompanied by an increase or decrease in tidal volume, which translates into an exponential variation in mechanical energy. In other words, as compliance decreases, ventilator-generated energy and airflow become uncoupled. Conversely, when applied to spontaneous breathing conditions, optimal coupling allows most respiratory pump energy to convert into airflow, rendering breathing more “efficient” (e.g., without ineffective efforts). As such, iso-MP may predict long-term ventilator dependence or autonomic breathing based on whether it is generated by high pressures along with low tidal volumes (consistent with high energy density and low compliance) or low pressures and high tidal volumes (low energy density at high compliance), referring to the degree of (un)coupling between energy and airflows.

MP density was significantly more accurate than traditional indexes (e.g., minute ventilation, VT/PBW, RSBI, or IWI) in predicting weaning outcomes, most likely because it better reflects prolonged weaning failure`s underlying pathophysiology, apparently an excessive respiratory muscle effort for carbon dioxide elimination. This aligns with previous studies on MP and weaning, in which MP density (e.g., LTC_dyn_-MP) has consistently demonstrated the most robust predictive ability regarding failure to wean^6,7,20^. This index incorporates respiratory rate and airway pressures, factors that also have been independently associated with weaning failure in the recent multicenter WEAN SAFE study^21^. MP density has also been shown to be an accurate predictor of prolonged ventilation after double lung transplantation, with LTC_dyn_-MP demonstrating the strongest correlation with invasive ventilation time following a patient’s transfer from the operating room to the ICU^22^. However, further research is required as other studies have demonstrated conflicting findings^23^.

IWI showed reasonable diagnostic accuracy, averaging between RSBI and dynamic respiratory system compliance, which are factored into this index. Interestingly, high VT/PBW was associated with sustained autonomic breathing, unlike clinical settings involving acute respiratory failures, where higher values predict escalating respiratory treatment (e.g., to invasive ventilation)^24^. This index probably reflects different pathophysiological mechanisms in acute and chronic respiratory failure, indicating a high respiratory drive in the acute setting^25^ but increased lung capacity during prolonged weaning. Similarly, a recent study of patients with non-cardiac thoracic surgery revealed an increased risk of postoperative pulmonary complications (e.g., respiratory failure) that were independently associated with a lower VT/PBW and a higher MP in conjunction with reduced dynamic respiratory system compliance^26^.

Regarding RSBI, this index has been developed to test a mechanically ventilated patient’s capacity to tolerate unassisted breathing^17^. Individuals who cannot tolerate a weaning trial typically develop a breathing pattern characterized by a high respiratory rate and low tidal volume immediately after disconnecting from the ventilator^27^. A limited number of studies examined the RSBI in the context of prolonged weaning, revealing poor diagnostic accuracy in predicting weaning outcomes^3,4,28^. Based on the MP density concept, reducing tidal volume will cause muscle stress to decline exponentially, whereas increasing respiratory rate will increase stress intensity linearly. Given this, a likely explanation for developing a rapid and shallow breathing pattern in general (e.g., during acute respiratory failure)^29^ is that it imposes less respiratory muscle stress (reducing the oxygen cost of breathing) and protects the respiratory pump against (fatal) failure. Despite RSBI being higher in weaning failures in the present study, the threshold that discriminated best between groups (61 min^−1^ * L^−1^) was lower compared to prior studies of intubated patients^17^. This observation may indicate differences in fatigue thresholds for acute respiratory distress (during a short weaning trial) and ventilatory failure (chronic hypercapnia), depending on the degree of stress intensity placed on respiratory muscles^30,31^. Accordingly, we found a significant correlation and independent relationship between MP density, dynamic compliance (mainly determining MP density), and RSBI, and this may also help explain why factors such as female gender and smaller endotracheal tube sizes (dynamic compliance is reduced due to smaller lung size and increased artificial airway resistance) are associated with higher RSBI^32^.

Although studies have not evaluated the relationship between MP or MP density and spontaneous breathing P_a_CO_2_, previous research has demonstrated compromised respiratory mechanics associated with increased respiratory variables used to calculate MP in hypercapnic patients failing SBT^27^. Since MP density demonstrated the strongest correlation with P_a_CO_2_ in the present study, this index may be a key determinant of autonomic breathing.

### Limitations

This study has limitations. Our computations for pressure-controlled ventilation were based on a simplified equation associated with a higher error rate than more comprehensive formulas^33^. Furthermore, ICU ventilators have significant inaccuracies in measuring pressures and flows compared to pressure transducers and flowmeters^34^, affecting breathing variables calculations. Another limitation is that no information is available regarding patients’ respiratory drive and inspiratory effort before and during SBT. Finally, we did not compare MP density with other measures of spontaneous work of breathing, such as esophageal manometry-derived tidal changes in esophageal pressure^25^, spontaneous mechanical power ratio and pressure-rate index (4DPRR)^35^, or changes in oxygen consumption during SBT^36^, which should be explored in future research.

## Conclusions

Several ventilatory and spontaneous breathing indexes readily available at the bedside can provide a comprehensive picture of a patient’s respiratory condition and assist clinicians in evaluating a patient’s spontaneous breathing ability following prolonged weaning. Study findings suggest the critical factor underlying sustained autonomic breathing is the mechanical stress intensity required for decarboxylation. MP density, based on thermodynamic principles, may help define and quantify stress intensity via less invasive, indirect methods, and it was more accurate at predicting weaning failures than established indexes assessed during spontaneous breathing.

### Supplementary Information

Supplementary Information.

## Data Availability

The datasets used and analyzed during the current study are available from the corresponding author upon reasonable request.
