# Supplementary material for: Mechanical power density, spontaneous breathing indexes, and prolonged weaning failure: a prospective cohort study
Source: Sci Rep. 2024 Jul 15;14:16297. doi: 10.1038/s41598-024-67237-w (PMC11251183; doi:10.1038/s41598-024-67237-w)
Supplement: Supplementary file 1 — Supplementary Information. [file 41598_2024_67237_MOESM1_ESM.pdf]

Mechanical power density, spontaneous breathing indexes, and prolonged weaning failure: a prospective cohort study

Alessandro Ghiani, MD; Swenja Walcher, RT; Azal Lutfi, MD; Joanna Paderewska, MD; Simon Ulrich Jaeger, MD; Nikolaus Kneidinger, MD; Stephanie Susanne Stecher, MD; Franziska Christina Trudzinski, MD; and Claus Neurohr, MD

Supplementary file 1

1. **Definitions of ventilatory indexes – Mechanical ventilation (Page 2)**
2. **Definitions of patients` spontaneous breathing indexes – CPAP breathing (Page 3)**
3. **Spontaneous breathing trial (SBT) protocol (Page 4)**
4. **Table S1: Results of prolonged weaning – Comparison of patients with weaning failure and success (Page 5)**
5. **Table S2: Start of weaning (first SBT) – Ventilatory & spontaneous breathing variables and indexes (Page 6)**
6. **Table S3: End of weaning (last SBT) – Ventilatory & spontaneous breathing variables and indexes (Page 7)**
7. **Table S4: Cross-validated performance of ventilatory and spontaneous breathing indexes at the end of weaning (last SBT) analyzed to predict prolonged weaning failure (Page 8)**
8. **Figure S1: Comparison of AUROC for MP density and LTC_{dyn} with spontaneous breathing indexes at the end of weaning (last SBT) (Page 9)**
9. **Table S5: Sensitivity analysis: Area under the ROC curve for each index analyzed to predict weaning failure at the start and end of weaning using a different P_aCO_2 threshold for ventilatory failure (> 50 mmHg) (Page 10)**
10. **Table S6: Sensitivity analysis: Area under the ROC curve for each index analyzed to predict weaning failure at the start and end of weaning in patients without COVID-19 pneumonia (N = 110) (Page 11)**
11. **Table S7: Correlations of ventilatory and spontaneous breathing indexes at last SBT with median spontaneous P_aCO_2 at the end of weaning (Page 12)**
12. **Table S8: Sensitivity analysis: Correlations of ventilatory and spontaneous breathing indexes at last SBT with median spontaneous P_aCO_2 at the end of weaning in weaning success patients (N = 99) (Page 13)**
13. **Table S9: Subgroup analysis – Comparison of patients with and without COPD (Page 14)**
14. **E-References (Page 15)**

Definitions of ventilatory indexes – Mechanical ventilation

**Ventilatory ratio (VR)**	Ventilatory ratio (VR) is a surrogate of pulmonary dead space fraction and a simple bedside index of impaired efficiency of ventilation[1-2]:<br><br>$$\mathbf{VR = VE_{measured} * P_aCO_{2\text{-}measured} / VE_{predicted} * P_aCO_{2\text{-}ideal}}$$<br><br>$VE_{measured}$ is the measured minute ventilation (mL/min), $P_aCO_{2\text{-}measured}$ is the measured arterial pressure of carbon dioxide (mmHg), $VE_{predicted}$ is the predicted minute ventilation calculated as predicted bodyweight x 100 (mL/min), and P_aCO2_{ideal} is the expected arterial pressure of carbon dioxide in normal lungs if ventilated with the predicted minute ventilation. $P_aCO_{2\text{-}ideal}$ is set at 37.5 mmHg (5 kPa) for all patients. VR is a unitless ratio, with a value approximating one representing normal ventilating lungs.
**Mechanical power (MP)**	Mechanical power (MP)[3] provided by the ventilator in the pressure-controlled mode was calculated using a simplified formula, including respiratory rate (RR), tidal volume (VT), and P_{max}[4-5]:<br><br>$$\mathbf{MP\ (Joule/min) = 0.098 * RR * VT * P_{max}}$$<br>$$\mathbf{MP\ (Joule/min) = 0.098 * VE * P_{max}}$$<br><br>With each breath the ventilator delivers, a certain amount of energy (Joule) is transferred to the patient's respiratory system. This energy is mainly used to overcome the airways' resistance, inflate the lungs, and expand the thoracic cage.
**Mechanical power normalized to dynamic lung-thorax compliance (LTC_{dyn}-MP)**	MP normalized to dynamic lung-thorax compliance (LTC_{dyn}-MP) was calculated using MP and dynamic lung-thorax compliance (LTC_{dyn})[6]:<br><br>$$\mathbf{LTC_{dyn}\text{-}MP\ (J/min * cmH_2O/mL) = MP / LTC_{dyn}}$$<br>$$\mathbf{LTC_{dyn}\text{-}MP\ (J/min * cmH_2O/mL) = (0.098 * RR * VT * P_{max}) * (\Delta P_{aw} / VT)}$$<br>$$\downarrow$$<br>$$\mathbf{LTC_{dyn}\text{-}MP\ (cmH_2O^2/min) = RR * P_{max} * \Delta P_{aw}}$$<br>$$\mathbf{LTC_{dyn}\text{-}MP\ (cmH_2O^2/min) = RR * P_{max} * (P_{max} - PEEP)}$$<br><br>This formula accounts for different effects of changes in respiratory rate, inflation pressure (P_{max}), and PEEP (and thus changes in ΔP_{aw}) on delivered energy. Increasing RR leads to a linear rise in energy transfer while increasing pressure (concomitantly increasing tidal volume) results in an exponential increment in power[3] and stress intensity.
**Power indexes of the respiratory system (PI_{rs})**	LTC_{dyn}-MP normalized to P_aCO_2 was calculated using LTC_{dyn}-MP and P_aCO_2 similarly to the corrected minute ventilation[6-7]:<br><br>$$\mathbf{PI_{rs}^X\ (cmH_2O^2/min) = LTC_{dyn}\text{-}MP * (P_aCO_{2\text{-}actual}/P_aCO_{2\text{-}target})^X}$$<br>$$\mathbf{PI_{rs}^X\ (cmH_2O^2/min) = RR * P_{max} * \Delta P_{aw} * (P_aCO_{2\text{-}actual}/P_aCO_{2\text{-}target})^X}$$<br><br>Since P_aCO_2 is inversely proportional to minute ventilation (neglecting dead space fraction), exponent X approximates values between 1 and 2, depending on whether adjustments of ventilator settings are made for RR and/or $P_{max}/\Delta P_{aw}$ to reach $P_aCO_{2\text{-}target}$, which was arbitrarily set at 45.0 mmHg (6.0 kPa, corresponding to the hypercapnic threshold) for all patients. Power $index_{rs}$ estimate the LTC_{dyn}-MP necessary for adequate alveolar ventilation (keeping P_aCO_2 below the hypercapnic threshold)[7].

Definitions of patients` spontaneous breathing indexes – CPAP breathing

**Tidal volume normalized to the predicted body weight (VT/PBW)**	Tidal volume was measured during the SBT, and we calculated PBW using the following equations[8]:<br><br>**PBW (males) = 50 + 0.91 * (body height [cm]–152.4)**<br>**PBW (females) = 45.5 + 0.91 * (body height [cm]–152.4)**
**Rapid shallow breathing index (RSBI)**	The rapid shallow breathing index, also known as the frequency-to-tidal volume ratio, is an index developed to test a mechanically ventilated patient's capacity to tolerate a trial of unassisted breathing[9] (referred to as readiness testing):<br><br>**RSBI (min^{-1} * L^{-1}) = RR / VT**<br><br>Individuals who cannot tolerate such a trial usually develop a breathing pattern characterized by a high respiratory rate and low tidal volume a few minutes after disconnecting from the ventilator, resulting in an increased RSBI. Weaning trial failure is predicted by a score > 105 in such patients[9].
**Integrative weaning index modified (IWI modified)**	The Integrative Weaning Index was developed to test a patient's capacity to tolerate a weaning trial and subsequent extubation[10]. We modified this index using dynamic instead of static respiratory system compliance for its computation:<br><br>**IWI modified (L^2/cmH_2O * % * min * 10^{-3}) = LTC_{dyn} * SaO_2 / RSBI**

Spontaneous breathing trial (SBT) protocol

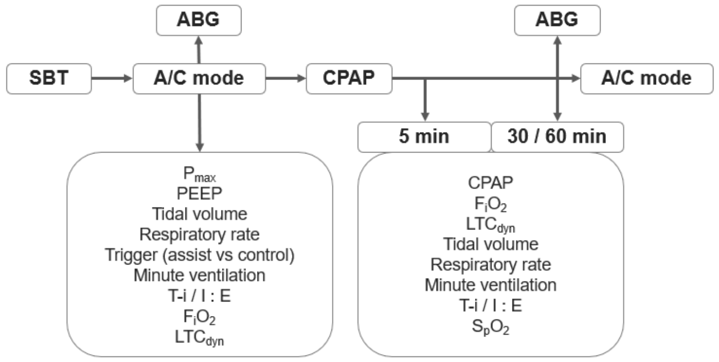

Patient - ID

Date/time of SBT: ________________ / ________________ First SBT (30 min) ☐ Last SBT (60 min) ☐

**A/C mode** (pre-SBT)			
P_{max} (cmH_2O)		Minute ventilation (L/min)	
PEEP (cmH_2O)		T_i (sec) / I:E	
Tidal volume (mL)		F_iO_2 (%)	
Respiratory rate (1/min)		LTC_{dyn} (mL/cmH_2O)	
Trigger	control ☐ assist ☐		

**CPAP**	**5 min**	**30 min (first) / 60 min (last)**
CPAP (cmH_2O)		
F_iO_2 (%)		
LTC_{dyn} (mL/cmH_2O)		
Tidal volume (mL)		
Respiratory rate (1/min)		
Minute ventilation (L/min)		
T_i (sec) / I:E		
S_pO_2 (%)		

**ABG**	**A/C mode – pre-SBT**	**CPAP – 30 min (first) / 60 min (last)**
P_aCO_2 (mmHg)		
P_aO_2 (mmHg)		
pH		
HCO_3 (mmol/L)		
S_aO_2 (%)		

**SBT result**	Success ☐	Failure ☐
Duration (min): ____________	**Hypercapnia** [$P_aCO_2 > 45.0$ mmHg]	yes ☐ no ☐
	**Respiratory acidosis** [pH < 7.35]	yes ☐ no ☐
	**Premature termination of SBT**	yes ☐ no ☐
Adapted from Boles et al. *Eur Respir J* 2007;29(5):1033-1056	**Physiological variables at the end of SBT:**<br>Respiratory rate: ________________ (1/min)<br>Blood pressure (sys/dia): ___________________ (mmHg)<br>Heart rate: ___________________ (bpm)	**Cause of premature termination:**<br>RR > 35/min ☐<br>Systolic blood pressure > 160 mmHg ☐<br>Tachycardia > 130 bpm ☐

Table S1: Results of prolonged weaning – Comparison of patients with weaning failure and success

**Results of prolonged weaning**	**All patients** (n = 140)	**Weaning failure** (n = 41)	**Weaning success** (n = 99)	***P* value**[a]
Weaning duration from first SBT (days)	13 (11–17)	15 (13–20)	13 (11–15)	**< 0.01**[b]
Duration of mechanical ventilation (days)[§]	41 (33–52)	44 (35–64)	40 (32–51)	0.136[b]
Weaning failure	41 (29)	41 (100)	–	–
Weaning success	99 (71)	–	99 (100)	–
Median P_aCO_2 at completion (mmHg)	40 (36–45)	51 (49–53)	38 (35–41)	–
Decannulation	71 (51)	14 (34)	57 (58)	**0.012**
Ventilator-attached at discharge	38 (27)	36 (88)	2 (2)	–
HMV-NIV	11 (8)	10 (25)	1 (1)	–
HMV-IMV	27 (19)	26 (63)	1 (1)	–
SB at weaning completion (hours per day)	24 (18–24)	16 (8–18)	24 (24–24)	–
LTOT at hospital discharge*	85 (61)	38 (93)	47 (49)	**< 0.01**[c]
Weaning unit-LOS (days)	28 (21–41)	36 (29–45)	25 (20–34)	**< 0.01**[b]

Legend

Continuous variables are presented as median (– interquartile range [IQR]); categorical variables are presented as numbers (%).

[§]: The total duration of mechanical ventilation, the period from intubation in the referring ICU until the completion of weaning at the weaning center.
[*]: Excluding three patients who died after weaning completion (after the last SBT)

a: P value for differences between the weaning failure and success group
b: Mann-Whitney *U*-test
c: Chi-squared test

Abbreviations: SBT, spontaneous breathing trial; HMV-NIV, Home mechanical ventilation – Non-invasive ventilation; HMV-IMV, Home mechanical ventilation – Invasive mechanical ventilation; SB, spontaneous breathing; LTOT, long-term oxygen therapy; LOS, length of stay

Table S2: Start of weaning (first SBT) – Ventilatory and spontaneous breathing variables and indexes

**Ventilatory variables and indexes** (pre-SBT)	**All patients** (n = 140)	**Weaning failure** (n = 41)	**Weaning success** (n = 99)	***P* value**[a]
PEEP (cmH_2O)	5 (5–5)	5 (5–5)	5 (5–5)	0.301[c]
Peak airway pressure (cmH_2O)	20 (20–21)	21 (20–23)	20 (20–21)	**< 0.01**[c]
Dynamic driving pressure (cmH_2O)	15 (15–16)	16 (15–18)	15 (15–16)	**< 0.01**[c]
P/F ratio (mmHg)	275 (228–332)	269 (225–297)	278 (231–343)	0.155[c]
Respiratory rate (min^{-1})	18 (16–23)	19 (16–23)	18 (16–23)	0.493[c]
Tidal volume (mL)	580 (506–684)	545 (473–623)	600 (518–719)	**0.016**[c]
VT/PBW (mL/kg)	8.8 (7.7–10.1)	8.2 (7.5–9.2)	9.1 (7.8–10.3)	0.079[c]
Minute ventilation (L/min)	10.8 (9.6–13.3)	10.2 (9.0–11.9)	11.1 (10.0–13.8)	**0.015**[c]
Mechanical ventilation P_aCO_2 (mmHg)	35 (30–39)	39 (35–43)	33 (28–38)	**< 0.01**[b]
Ventilatory ratio	1.51 (1.29–1.80)	1.65 (1.39–2.11)	1.40 (1.26–1.73)	**< 0.01**[c]
LTC_{dyn} (mL/cmH_2O)	38 (30–44)	32 (29–39)	39 (33–47)	**< 0.01**[b]
Mechanical power (Joule/min)	22.3 (18.8–27.0)	21.6 (17.8–26.3)	22.3 (19.1–27.4)	0.405[c]
MP density indexes				
LTC_{dyn}-MP (cmH_2O^2/min)	5712 (5054–7500)	6900 (5400–8529)	5400 (4800–7094)	**< 0.01**[c]
Power $index_{rs}^{1.0}$ (cmH_2O^2/min)	4552 (3573–6365)	5943 (4509–7858)	4224 (3271–5257)	**< 0.01**[c]
Power $index_{rs}^{2.0}$ (cmH_2O^2/min)	3560 (2520–5506)	5116 (3601–7020)	3004 (2034–4676)	**< 0.01**[c]
**Patients` breathing variables and indexes** (after 30 min of CPAP)	**All patients** (n = 140)	**Weaning failure** (n = 41)	**Weaning success** (n = 99)	***P* value**[a]
CPAP (cmH_2O)	5 (5–5)	5 (5–5)	5 (5–5)	0.248[c]
P/F ratio (mmHg)	265 (231–320)	247 (219–290)	273 (237–338)	**0.034**[c]
Respiratory rate (min^{-1})	26 (23–30)	26 (22–28)	26 (24–30)	0.929[c]
Tidal volume (mL)	436 (350–489)	350 (297–441)	452 (395–525)	**< 0.01**[c]
Minute ventilation (L/min)	11.0 (9.0–13.4)	9.7 (7.8–11.4)	11.7 (9.5–13.9)	**< 0.01**[b]
Spontaneous breathing P_aCO_2 (mmHg)	39 (34–45)	46 (42–51)	36 (32–43)	**< 0.01**[c]
VT/PBW (mL/kg)	6.5 (5.7–7.5)	5.8 (5.1–6.7)	6.6 (6.0–7.9)	**< 0.01**[c]
RSBI ($min^{-1}*L^{-1}$)	62 (49–78)	69 (54–97)	58 (43–73)	**< 0.01**[c]
IWI modified ($L^2/cmH_2O*\%*min*10^{-3}$)	58 (41–81)	43 (31–58)	62 (46–91)	**< 0.01**[c]

Legend

Continuous variables are presented as median (– interquartile range [IQR]).

a: P value for differences between the weaning failure and success group
b: Student's *t*-test
c: Mann-Whitney *U*-test

Abbreviations: SBT, spontaneous breathing trial; PEEP, positive end-expiratory pressure; P/F ratio, the ratio of partial pressure of oxygen to fraction of inspired oxygen; VT/PBW, tidal volume normalized to the predicted body weight; LTC_{dyn}, dynamic lung-thorax compliance; LTC_{dyn}-MP, mechanical power normalized to dynamic lung-thorax compliance; CPAP, continuous positive airway pressure; RSBI, rapid-shallow breathing index; IWI modified, modified integrative weaning index.

Table S3: End of weaning (last SBT) – Ventilatory and spontaneous breathing variables and indexes

**Ventilatory variables and indexes** (pre-SBT)	**All patients** (n = 140)	**Weaning failure** (n = 41)	**Weaning success** (n = 99)	***P* value**[a]
PEEP (cmH_2O)	5 (5–5)	5 (5–5)	5 (5–5)	0.928[c]
Peak airway pressure (cmH_2O)	20 (20–21)	21 (20–23)	20 (19–21)	**< 0.01**[c]
Dynamic driving pressure (cmH_2O)	15 (15–16)	16 (15–18)	15 (14–15)	**< 0.01**[c]
P/F ratio (mmHg)	286 (255–336)	267 (231–298)	293 (260–359)	**< 0.01**[c]
Respiratory rate (min^{-1})	18 (16–21)	18 (17–22)	18 (16–21)	0.199[c]
Tidal volume (mL)	566 (486–687)	500 (435–594)	588 (508–712)	**< 0.01**[c]
VT/PBW (mL/kg)	8.8 (7.5–10.1)	7.9 (6.7–9.3)	9.2 (7.9–10.2)	**0.012**[c]
Minute ventilation (L/min)	10.7 (9.0–12.7)	10.1 (8.6–11.2)	11.1 (9.1–12.9)	**0.016**[c]
Mechanical ventilation P_aCO_2 (mmHg)	36 (31–39)	42 (38–46)	33 (30–37)	**< 0.01**[b]
Ventilatory ratio	1.51 (1.32–1.78)	1.76 (1.46–1.99)	1.49 (1.25–1.67)	**< 0.01**[c]
LTC_{dyn} (mL/cmH_2O)	36 (31–45)	29 (24–35)	39 (33–48)	**< 0.01**[c]
Mechanical power (Joule/min)	21.6 (17.5–25.3)	21.6 (17.7–25.3)	21.6 (17.2–25.3)	0.996[c]
MP density indexes				
LTC_{dyn}-MP (cmH_2O^2/min)	5700 (4857–7056)	7038 (5928–8310)	5100 (4800–6344)	**< 0.01**[c]
Power $index_{rs}^{1.0}$ (cmH_2O^2/min)	4538 (3462–5706)	6554 (5104–8250)	4011 (3290–5109)	**< 0.01**[c]
Power $index_{rs}^{2.0}$ (cmH_2O^2/min)	3691 (2604–4906)	6156 (4402–7910)	3004 (2153–3917)	**< 0.01**[c]
**Patients` breathing variables and indexes** (after 60 min of CPAP)	**All patients** (n = 140)	**Weaning failure** (n = 41)	**Weaning success** (n = 99)	***P* value**[a]
CPAP (cmH_2O)	5 (5–5)	5 (5–5)	5 (5–5)	0.193[c]
P/F ratio (mmHg)	312 (259–362)	269 (220–313)	326 (279–370)	**< 0.01**[b]
Respiratory rate (min^{-1})	25 (20–29)	25 (19–30)	24 (20–29)	0.644[b]
Tidal volume (mL)	430 (360–490)	380 (312–439)	445 (386–532)	**< 0.01**[c]
Minute ventilation (L/min)	10.2 (8.2–12.6)	9.0 (7.3–10.2)	11.4 (8.5–13.3)	**< 0.01**[c]
Spontaneous breathing P_aCO_2 (mmHg)	39 (34–44)	46 (43–51)	37 (33–41)	**< 0.01**[b]
VT/PBW (mL/kg)	6.3 (5.6–7.8)	5.8 (4.8–6.8)	6.6 (5.7–7.9)	**< 0.01**[c]
RSBI ($min^{-1}*L^{-1}$)	58 (42–77)	68 (44–91)	55 (41–76)	**0.032**[c]
IWI modified ($L^2/cmH_2O*\%*min*10^{-3}$)	64 (40–95)	41 (25–72)	71 (50–106)	**< 0.01**[c]

Legend

Continuous variables are presented as median (– interquartile range [IQR]).

a: P value for differences between the weaning failure and success group
b: Student's *t*-test
c: Mann-Whitney *U*-test

Abbreviations: SBT, spontaneous breathing trial; PEEP, positive end-expiratory pressure; P/F ratio, the ratio of partial pressure of oxygen to fraction of inspired oxygen; VT/PBW, tidal volume normalized to the predicted body weight; LTC_{dyn}, dynamic lung-thorax compliance; LTC_{dyn}-MP, mechanical power normalized to dynamic lung-thorax compliance; CPAP, continuous positive airway pressure; RSBI, rapid-shallow breathing index; IWI modified, modified integrative weaning index.

Table S4: Cross-validated performance of ventilatory and spontaneous breathing indexes at the end of weaning (last SBT) analyzed to predict prolonged weaning failure

**Ventilatory indexes** (threshold)	**Sensitivity**	**Specificity**	**PPV**	**NPV**	**Accuracy**	**PLR**	**NLR**	**DOR**	**F_1**	**MCC**
Ventilatory ratio (1.69)	48 (16-81)	70 (47-87)	42 (20-67)	76 (60-86)	64 (44-81)	1.9 (0.6-6.6)	0.8 (1.6-0.4)	3.1	0.43	0.18
LTC_{dyn} (34 mL/cmH_2O)	65 (30-90)	71 (47-88)	50 (28-69)	83 (64-92)	69 (49-84)	3.0 (1.0-12)	0.5 (1.3-0.2)	8.6	0.54	0.34
Mechanical power (26.5 Joule/min)	22 (5-60)	75 (53-88)	30 (8-69)	68 (57-77)	58 (39-76)	1.2 (0.5-11)	1.1 (2.4-0.7)	1.3	0.22	-0.02
LTC_{dyn}-MP (5965 cmH_2O^2/min)	70 (31-93)	66 (42-86)	47 (29-68)	83 (63-93)	66 (47-83)	2.4 (1.0-6.4)	0.5 (1.3-0.4)	7.1	0.55	0.33
Power $index_{rs}^{1.0}$ (4714 cmH_2O^2/min)	85 (47-99)	70 (47-88)	57 (38-73)	91 (68-98)	75 (55-89)	3.2 (1.4-7.9)	0.2 (1.1-0.1)	16.4	0.67	0.51
Power $index_{rs}^{2.0}$ (4594 cmH_2O^2/min)	70 (33-92)	84 (63-95)	70 (39-85)	86 (69-93)	79 (61-91)	9.1 (1.7-55)	0.4 (1.1-0.2)	48.0	0.68	0.55
**Spontaneous breathing indexes** (threshold)	**Sensitivity**	**Specificity**	**PPV**	**NPV**	**Accuracy**	**PLR**	**NLR**	**DOR**	**F_1**	**MCC**
VT/PBW (6.1 mL/kg)	63 (25-91)	62 (38-82)	42 (24-61)	80 (60-91)	62 (42-81)	1.8 (0.8-4.1)	0.6 (1.7-0.2)	3.2	0.49	0.23
RSBI (61 min^{-1}*L^{-1})	63 (26-91)	61 (37-81)	43 (25-61)	80 (59-91)	62 (42-79)	2.1 (0.8-5.9)	0.7 (1.8-0.3)	4.2	0.50	0.23
IWI modified (50 L^2/cmH_2O*%*min*10^{-3})	55 (20-86)	73 (49-89)	49 (26-71)	80 (63-90)	68 (48-84)	2.5 (0.8-8.5)	0.6 (1.5-0.6)	4.4	0.50	0.28

Legend

Results of 2-times repeated, 5-fold cross-validation. Mean metrics of diagnostic accuracy (with 95% confidence intervals) based on threshold values associated with the *Youden index* (presented as the mean of the thresholds derived from the training sets).

Abbreviations: PPV, positive predictive value; NPV, negative predictive value; PLR, positive likelihood ratio; NLR, negative likelihood ratio; DOR, diagnostic odds ratio; F_1, F_1 score; MCC, Matthews` correlation coefficient; VR, ventilatory ratio; LTC_{dyn}, dynamic lung-thorax compliance; LTC_{dyn}-MP, mechanical power normalized to dynamic lung-thorax compliance; VT/PBW, tidal volume normalized to the predicted body weight; RSBI, rapid shallow breathing index, IWI modified, modified integrative weaning index.

Figure S1: Comparison of AUROC for MP density and LTC_{dyn} with spontaneous breathing indexes at the end of weaning (last SBT)

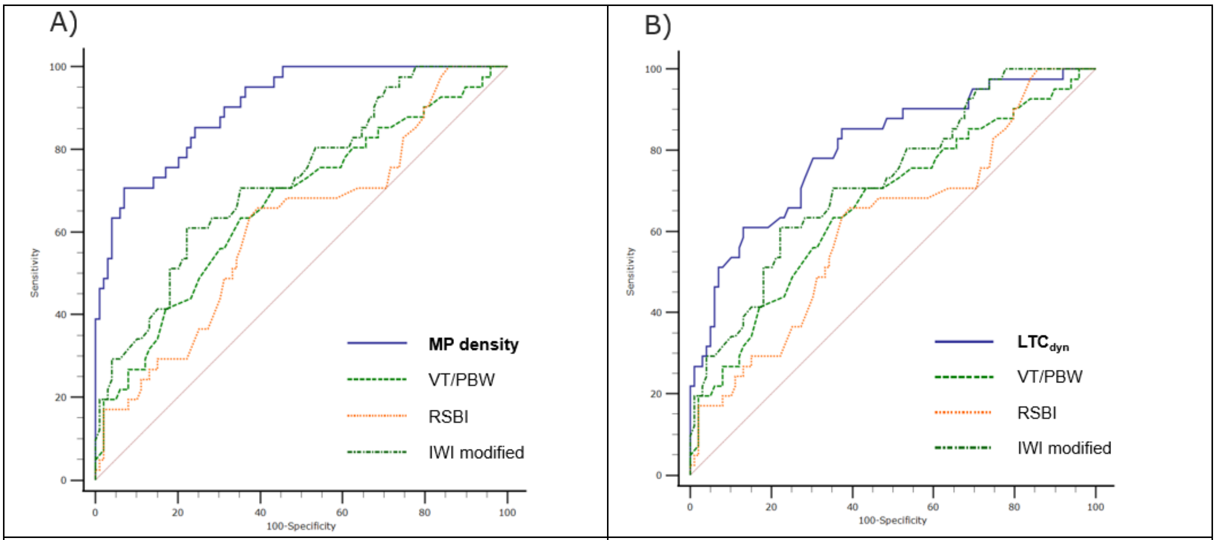

Comparison of the MP density and		Comparison of LTC_{dyn} and	
**1. VT/PBW**		**1. VT/PBW**	
Difference between areas	0.238	Difference between areas	0.138
95%CI	0.135 – 0.342	95%CI	0.026 – 0.249
z statistic	4.514	z statistic	2.420
P value	< 0.01	*P* value	0.016
**2. RSBI**		**2. RSBI**	
Difference between areas	0.289	Difference between areas	0.189
95%CI	0.177 – 0.401	95%CI	0.079 – 0.298
z statistic	5.060	z statistic	3.379
P value	< 0.01	*P* value	< 0.01
**3. IWI modified**		**3. IWI modified**	
Difference between areas	0.177	Difference between areas	0.077
95%CI	0.087 – 0.268	95%CI	0.007 – 0.146
z statistic	3.839	z statistic	2.165
P value	< 0.01	*P* value	0.030

Legend: Comparison of AUROC for MP density (expressed as Power $index_{rs}^{2.0}$) (Panel A) and LTC_{dyn} (Panel B) with spontaneous breathing indexes at the end of weaning (last SBT)

Abbreviations: AUROC, area under the receiver operating characteristic curve; MP, mechanical power; LTC_{dyn}, dynamic lung-thorax compliance; VT/PBW, tidal volume normalized to the predicted body weight; RSBI, rapid shallow breathing index; IWI modified, modified Integrative weaning index, 95%CI, 95% confidence interval.

Table S5: Sensitivity analysis: Area under the ROC curve for each index analyzed to predict weaning failure at the start and end of weaning using a different P_aCO_2 threshold for ventilatory failure (> 50 mmHg)

**Ventilatory indexes** (pre-SBT)	**Start of weaning** (first SBT)	***P* value**	**End of weaning** (last SBT)	***P* value**
Ventilatory ratio	0.69 (0.61 – 0.77)	**< 0.01**	0.67 (0.59 – 0.75)	**< 0.01**
LTC_{dyn} (mL/cmH_2O)	0.71 (0.63 – 0.78)	**< 0.01**	0.79 (0.72 – 0.86)	**< 0.01**
Mechanical power (Joule/min)	0.50 (0.42 – 0.59)	0.968	0.53 (0.44 – 0.61)	0.669
MP density indexes				
LTC_{dyn}-MP (cmH_2O^2/min)	0.69 (0.61 – 0.77)	**< 0.01**	0.76 (0.69 – 0.83)	**< 0.01**
Power $index_{rs}^{1.0}$ (cmH_2O^2/min)	0.73 (0.65 – 0.80)	**< 0.01**	0.86 (0.79 – 0.91)	**< 0.01**
Power $index_{rs}^{2.0}$ (cmH_2O^2/min)	0.75 (0.66 – 0.82)	**< 0.01**	0.89 (0.82 – 0.94)	**< 0.01**
**Patients` breathing indexes** (after 30/60 min)	**Start of weaning** (first SBT)	***P* value**	**End of weaning** (last SBT)	***P* value**
VT/PBW (mL/kg)	0.73 (0.65 – 0.80)	**< 0.01**	0.62 (0.54 – 0.70)	0.068
RSBI (min^{-1}*L^{-1})	0.73 (0.65 – 0.80)	**< 0.01**	0.58 (0.49 – 0.66)	0.246
IWI modified (L^2/cmH_2O*%*min*10^{-3})	0.76 (0.69 – 0.83)	**< 0.01**	0.69 (0.61 – 0.74)	**< 0.01**

Legend

The accuracy of each ventilatory variable in predicting weaning failure is presented as the area under the ROC curve with 95% confidence intervals. The redefined weaning failure criterion (P_aCO_2 > 50 mmHg) was met by 24 (17%) out of 140 patients.

Abbreviations: SBT, spontaneous breathing trial; P/F ratio, the ratio of partial pressure of oxygen to fraction of inspired oxygen; LTC_{dyn}, dynamic lung-thorax compliance; LTC_{dyn}-MP, mechanical power normalized to dynamic lung-thorax compliance; VT/PBW, tidal volume normalized to the predicted body weight; RSBI, rapid shallow breathing index; IWI modified, modified integrative weaning index.

Table S6: Sensitivity analysis: Area under the ROC curve for each index analyzed to predict weaning failure at the start and end of weaning in patients without COVID-19 pneumonia (N = 110)

**Ventilatory indexes** (pre-SBT)	**Start of weaning** (first SBT)	***P* value**	**End of weaning** (last SBT)	***P* value**
Ventilatory ratio	0.71 (0.61 – 0.79)	**< 0.01**	0.73 (0.64 – 0.81)	**< 0.01**
LTC_{dyn} (mL/cmH_2O)	0.69 (0.60 – 0.78)	**< 0.01**	0.81 (0.72 – 0.88)	**< 0.01**
Mechanical power (Joule/min)	0.53 (0.43 – 0.62)	0.673	0.56 (0.46 – 0.65)	0.312
MP density indexes				
LTC_{dyn}-MP (cmH_2O^2/min)	0.68 (0.59 – 0.77)	**< 0.01**	0.83 (0.75 – 0.90)	**< 0.01**
Power $index_{rs}^{1.0}$ (cmH_2O^2/min)	0.77 (0.68 – 0.84)	**< 0.01**	0.91 (0.84 – 0.95)	**< 0.01**
Power $index_{rs}^{2.0}$ (cmH_2O^2/min)	0.80 (0.71 – 0.87)	**< 0.01**	0.92 (0.85 – 0.96)	**< 0.01**
**Patients` breathing indexes** (after 30/60 min)	**Start of weaning** (first SBT)	***P* value**	**End of weaning** (last SBT)	***P* value**
VT/PBW (mL/kg)	0.67 (0.57 – 0.76)	**< 0.01**	0.66 (0.56 – 0.74)	**< 0.01**
RSBI ($min^{-1}*L^{-1}$)	0.63 (0.53 – 0.72)	**0.030**	0.65 (0.56 – 0.74)	**< 0.01**
IWI modified ($L^2/cmH_2O*\%*min*10^{-3}$)	0.70 (0.61 – 0.79)	**< 0.01**	0.77 (0.68 – 0.84)	**< 0.01**

Legend

The accuracy of each ventilatory variable in predicting weaning failure is presented as the area under the ROC curve with 95% confidence intervals. There were 35 out of 110 patients (32%) who failed prolonged weaning and remained ventilator dependent.

Abbreviations: SBT, spontaneous breathing trial; P/F ratio, the ratio of partial pressure of oxygen to fraction of inspired oxygen; LTC_{dyn}, dynamic lung-thorax compliance; LTC_{dyn}-MP, mechanical power normalized to dynamic lung-thorax compliance; VT/PBW, tidal volume normalized to the predicted body weight; RSBI, rapid shallow breathing index; IWI modified, modified integrative weaning index.

Table S7: Correlations of ventilatory and spontaneous breathing indexes at last SBT with median spontaneous P_aCO_2 at the end of weaning

**Ventilatory indexes** (pre-SBT)	**Spearmans` correlation coefficient (*ρ*)**	***P* value**
Ventilatory ratio	0.36 (0.20 – 0.49)	**< 0.01**
LTC_{dyn} (mL/cmH_2O)	–0.45 (–0.57 – –0.31)	**< 0.01**
Mechanical power (Joule/min)	–0.04 (–0.21 – 0.13)	0.620
MP density indexes		
LTC_{dyn}-MP (cmH_2O^2/min)	0.42 (0.27 – 0.55)	**< 0.01**
Power $index_{rs}^{1.0}$ (cmH_2O^2/min)	0.66 (0.55 – 0.74)	**< 0.01**
Power $index_{rs}^{2.0}$ (cmH_2O^2/min)	0.73 (0.64 – 0.80)	**< 0.01**
**Patients` breathing indexes** (after 60 min)	**Spearmans` correlation coefficient (*ρ*)**	***P* value**
VT/PBW (mL/kg)	–0.22 (–0.38 – –0.06)	**< 0.01**
RSBI ($min^{-1}*L^{-1}$)	0.09 (–0.08 – 0.25)	0.292
IWI modified ($L^2/cmH_2O*\%*min*10^{-3}$)	–0.28 (–0.43 – –0.12)	**< 0.01**

Legend: Spearmans` correlation coefficient (*ρ*) with 95% confidence intervals.

Abbreviations: SBT, spontaneous breathing trial; LTC_{dyn}, dynamic lung-thorax compliance; LTC_{dyn}-MP, mechanical power normalized to dynamic lung-thorax compliance; VT/PBW, tidal volume normalized to the predicted body weight; RSBI, rapid shallow breathing index; IWI modified, modified Integrative weaning index.

Table S8: Sensitivity analysis: Correlations of ventilatory and spontaneous breathing indexes at last SBT with median spontaneous P_aCO_2 at the end of weaning in weaning success patients (N = 99)

**Ventilatory indexes**	**Spearmans` correlation coefficient (*ρ*)**	***P* value**
Ventilatory ratio (unitless)	0.20 (–0.00 – 0.38)	0.050
LTC_{dyn} (mL/cmH_2O)	–0.19 (–0.37 – 0.01)	0.063
Mechanical power (Joule/min)	–0.04 (–0.24 – 0.16)	0.682
MP density indexes		
LTC_{dyn}-MP (cmH_2O^2/min)	0.17 (–0.03 – 0.36)	0.090
Power $index_{rs}^{1.0}$ (cmH_2O^2/min)	0.46 (0.29 – 0.60)	**< 0.01**
Power $index_{rs}^{2.0}$ (cmH_2O^2/min)	0.55 (0.39 – 0.67)	**< 0.01**
**Patients` breathing indexes**	**Spearmans` correlation coefficient (*ρ*)**	***P* value**
VT/PBW (mL/kg)	–0.08 (–0.28 – 0.11)	0.404
RSBI ($min^{-1}*L^{-1}$)	–0.08 (–0.27 – 0.12)	0.429
IWI modified ($L^2/cmH_2O*\%*min*10^{-3}$)	–0.04 (–0.24 – 0.16)	0.678

Legend: Spearmans` correlation coefficient (*ρ*) with 95% confidence intervals.

Abbreviations: SBT, spontaneous breathing trial; LTC_{dyn}, dynamic lung-thorax compliance; LTC_{dyn}-MP, mechanical power normalized to dynamic lung-thorax compliance; VT/PBW, tidal volume normalized to the predicted body weight; RSBI, rapid shallow breathing index; IWI modified, modified Integrative weaning index.

Table S9: Subgroup analysis – Comparison of patients with and without COPD

**Clinical characteristics**	**All patients** (n = 140)	**COPD** (n = 25)	**Non-COPD** (n = 115)	***P* value**[a]
Age (years)	68 (59–74)	69 (60–73)	68 (57–74)	0.777[b]
Female gender	48 (34)	10 (40)	38 (33)	0.508[c]
APACHE-II (points)	15 (11–17)	15 (14–18)	14 (11–17)	0.128[b]
Ventilator days on admission	23 (16–34)	20 (14–28)	23 (16–36)	0.120[b]
Intubation to tracheostomy (days)	13 (9–18)	13 (7–16)	13 (9–18)	0.273[b]
**Ventilatory variables & indexes** (pre-last SBT)				
P/F ratio (mmHg)	286 (255–336)	280 (238–311)	286 (259–342)	0.228[b]
VT/PBW (mL/kg)	8.8 (7.5–10.1)	8.3 (7.4–9.8)	9.0 (7.5–10.1)	0.570[b]
Mechanical ventilation P_aCO_2 (mmHg)	36 (31–39)	39 (36–46)	35 (31–38)	**< 0.01**[b]
Ventilatory ratio	1.51 (1.32–1.78)	1.52 (1.46–1.86)	1.50 (1.29–1.76)	0.130[b]
LTC_{dyn} (mL/cmH_2O)	36 (31–45)	31 (28–38)	37 (32–46)	**< 0.01**[b]
Mechanical power (Joule/min)	21.6 (17.5–25.3)	21.2 (19.4–25.4)	21.6 (17.1–25.3)	0.553[b]
LTC_{dyn}-MP (cmH_2O^2/min)	5700 (4857–7056)	7056 (5100–8226)	5400 (4800–6696)	**< 0.01**[b]
Power $index_{rs}^{2.0}$ (cmH_2O^2/min)	3691 (2604–4906)	5586 (3784–7525)	3389 (2393–4518)	**< 0.01**[b]
**Patients` breathing variables & indexes** (after 60 min of CPAP at last SBT)				
Spontaneous breathing P_aCO_2 (mmHg)	39 (34–44)	46 (42–52)	38 (34–42)	**< 0.01**[b]
VT/PBW (mL/kg)	6.3 (5.6–7.8)	6.0 (4.8–6.6)	6.5 (5.6–7.9)	0.076[b]
RSBI ($min^{-1}*L^{-1}$)	58 (42–77)	61 (44–81)	57 (42–77)	0.358[b]
IWI modified ($L^2/cmH_2O*\%*min*10^{-3}$)	64 (40–95)	53 (31–70)	65 (41–97)	**0.032**[b]
**Results of prolonged weaning**				
Weaning duration from first SBT (days)	13 (11–17)	14 (11–17)	13 (11–18)	0.768[b]
Duration of mechanical ventilation (days)[§]	41 (33–52)	41 (33–49)	40 (33–55)	0.562[b]
Weaning failure	41 (29)	18 (72)	23 (20)	**< 0.01**[c]
Weaning unit-LOS (days)	28 (21–41)	33 (25–44)	27 (21–39)	0.050[b]

Legend

Continuous variables are presented as median (– interquartile range [IQR]); categorical variables are presented as numbers (%).

[§]: The total duration of mechanical ventilation, the period from intubation in the referring ICU until the completion of weaning at the weaning center.

a: P value for differences between patients with and without COPD
b: Mann-Whitney *U*-test
c: Chi-squared test

Abbreviations: COPD, chronic obstructive pulmonary disease; APACHE-II, Acute Physiology and Chronic Health Evaluation II score; SBT, spontaneous breathing trial; P/F ratio, the ratio of partial pressure of oxygen to fraction of inspired oxygen; VT/PBW, tidal volume normalized to the predicted body weight; LTC_{dyn}, dynamic lung-thorax compliance; LTC_{dyn}-MP, mechanical power normalized to dynamic lung-thorax compliance; RSBI, rapid-shallow breathing index; IWI modified, modified integrative weaning index; LOS, length of stay.

E-References

1. Sinha P, et al. Ventilatory ratio: a simple bedside measure of ventilation. *Br J Anaesth* 2009;102:692–697
2. Sinha P, et al. Analysis of ventilatory ratio as a novel method to monitor ventilatory adequacy at the bedside. *Crit Care* 2013;17:R34
3. Gattinoni L, et al. Ventilator-related causes of lung injury: the mechanical power. *Intensive Care Med* 2016;42:1567–1575
4. Becher T, et al. Calculation of mechanical power for pressure-controlled ventilation. *Intensive Care Med* 2019;45:1321–1323
5. Chiumello D, et al. Bedside calculation of mechanical power during volume- and pressure-controlled mechanical ventilation. *Crit Care* 2020;24:417
6. Ghiani A, et al. Mechanical power normalized to lung-thorax compliance predicts prolonged ventilation weaning failure: a prospective study. *BMC Pulmonary Medicine* 2021;21:202
7. Wexler HR & Lok P. A simple formula for adjusting arterial carbon dioxide tension. *Can Anaesth Soc J*. 1981;28(4):370–372.
8. Brower RG, et al. Ventilation with lower tidal volumes as compared with traditional tidal volumes for acute lung injury and the acute respiratory distress syndrome. *N Engl J Med* 2000;342: 1301–1308
9. Yang KL & Tobin MJ. A prospective study of indexes predicting the outcome of trials of weaning from mechanical ventilation. *N Engl J Med* 1991;324(21):1445–1450
10. Nemer SN, et al. A new integrative weaning index of discontinuation from mechanical ventilation. *Crit Care* 2009;13:R152
